# Jaws can be referred to as narrow or hypoplastic, but the term “atresia” is inaccurate!

**DOI:** 10.1590/2177-6709.23.5.019-023.oin

**Published:** 2018

**Authors:** Alberto Consolaro, Renata Bianco Consolaro

**Affiliations:** 1 Universidade de São Paulo, Faculdade de Odontologia de Bauru (Bauru/SP, Brazil).; 2 Universidade de São Paulo, Faculdade de Odontologia de Ribeirão Preto, Programa de Pós-graduação em Odontopediatria (Ribeirão Preto/SP, Brazil).; 3 Centro Universitário de Adamantina (Adamantina/SP, Brazil).

**Keywords:** Atresia, Atresic maxilla, Atrophy, Hypoplasia, Micrognathia

## Abstract

In order to lead to insights and discussion on proper use of Orthodontics and Pathology-related terminology, particularly in cases of smaller-than-usual maxilla and mandible - that is, anomalous ones -, this study compared the conceptual meaning of the term “atresia.” It is considered improper when referring to maxilla and mandible with deficient growth compared to development that is satisfactory enough to reach normal size. To identify smaller maxilla and mandible, the most proper and accurate term is hypoplastic maxilla or mandible. This is because “atresia” stands for an anomaly related to lumen blockage in hollow organs, which is not the case for neither maxilla nor mandible. Hypoplastic maxilla or mandible can be properly and specifically referred to as micrognathia.

## RESUMO

Para induzir reflexões e discussões sobre o uso adequado da nomenclatura em Ortodontia e Patologia, para os casos em que a maxila e a mandíbula apresentam-se pequenas ou menores do que o habitual, ou seja, anômalas, comparou-se o significado conceitual do termo “atrésica”. Esse termo não é adequado quando aplicado à maxila e à mandíbula para identificar situações em que houve um desenvolvimento com crescimento insuficiente para se chegar ao tamanho normal. Para identificar maxila e mandíbula menores, é mais adequado e preciso o uso do termo maxila ou mandíbula hipoplásica. Isso porque atresia representa uma anomalia por obstrução da luz ou lume em órgãos ocos, o que não ocorre na maxila ou na mandíbula. Maxila ou mandíbula hipoplásica também podem ser chamadas, apropriada e especificamente, de micrognatia.

Developmental disorders are changes occurring during one’s growth. Development largely persists after birth until the period during which the human body is formed ceases.^1^ ^-^ [Bibr B1] [Bibr B2] [Bibr B3] [Bibr B4] [Bibr B5] [Bibr B6] [Bibr B7] [Bibr B8] [Bibr B9] [Bibr B10] [Bibr B11] [Bibr B12] [Bibr B13] ^14^ In Dentistry, consensus has been reached on the fact that maxillary development, oftentimes known as growth, stops at around 22-24 years of age. Nevertheless, this varies among individuals, particularly when females and males are compared.

## WHEN IS IT THE CASE OF ANOMALY, DYSPLASIA OR DEFORMITY? 

**Figure 1:**
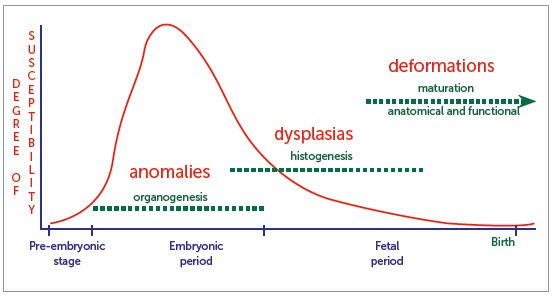
Periods of human development (in blue), respective predominant phenomena (in green), and groups of developmental disorders (in red) occurring upon action of teratogenic agents. Red curve signals the degree of susceptibility and risk involved in each period.

Human development (Fig 1) encompasses three distinct stages: 

**1** ^**st**^ **) Pre-embryonic stage:** 13 to 21 days after fertilization, when the process of gastrulation occurs, embryonic cells undergo differentiation and organize themselves into three distinct populations also known as germ layers: ectoderm, mesoderm, and endoderm. During this period, it is all or nothing: should any teratogenic agent act, the consequence is miscarriage; the pre-embryo hardly ever survives.
**2** ^**nd**^ **) Embryonic period:** it is the most critical phase, being susceptible to the action of both inner and outer agents for the following reasons: 1. It is characterized by numerous mitosis and cell differentiation processes; and 2. It is concluded by the end of the third month when most women don’t even know they are pregnant.

During this stage, organs and tissues are maturing, with a great deal of cell mobilization - which, altogether, is known as organogenesis. Anomalies or malformations are the names given to developmental disorders happening at this stage, significantly compromising tissues and organs in number, size and shape. 

Anomalies can be minor when affecting only one tissue or organ. However, the causing agent might act in several tissues and parts at the same time, thus leading to multiple anomalies, classified as syndromes, associations or sequences, depending on the criteria.

From both morphological and conceptual perspectives, anomalies or malformations might be caused by: 1) Incomplete development of an organ or tissue; 2) Redundancy; or 3) Aberration. Examples of a few anomalies caused by incomplete development of an organ or tissue are as follows:

» Agenesis: the utmost in malformations, it is characterized by complete absence of development of an organ, such as renal agenesis and anodontia.
» Aplasia: it is characterized by formation of the rudiments of organs which are completely unfunctional. From a physiological perspective, aplasia has the same effect of agenesis.
» Hypoplasia: consists of deficient development of an organ; however, without completely damaging function. Those organs are deficient in size, as it is the case of renal hypoplasia, producing kidneys deficient in structure, but functional. Enamel hypoplasia reveals a reduction in this tissue quantity and quality. Mandibular hypoplasia results in mandibles that are reduced in size, but that allow individuals to live a quite normal life. It can be corrected by surgical, orthopedic and/or orthodontic treatment.
» Atresia: deficiency or lack of development of lumen present in hollow organs, such as the digestive tube, trachea and excretory ducts, of which embryonic outlines were previously solid.
» Stenosis: specific narrowing of hollow structures, such as ducts, lumina or natural openings. As a result, the functional disorder will comprise partial obstruction.
» Persistence: failure of transient embryonic structures to regress, as it is the case of Meckel’s diverticulum, thyroglossal duct and dental lamina. Remnants or persistent vestigial structures, shaped as clusters or tissue fragments, might produce undesirable lesions, such as thyroglossal duct cyst, as well as odontogenic cysts and neoplasm.

**3** ^**rd**^ **) Fetal period:** from third month to birth, tissues begin a process of structural maturation and functional capacitation also known as histogenesis (Fig 1). At the beginning of the fetal period, the developing individual resembles a human being in shape and, for this reason, is identified as fetus. Before that, it is referred to as embryo which has not yet acquired human shape.

Should any alterations occur during this period, tissues previously formed are affected, particularly in terms of organizational maturation and functional capacity. Such alterations are known called dysplasias. Fibrous dysplasia of the maxilla, for instance, is characterized by tissue that is formed in an unorganized manner, with functional difficulty to fulfill the mission of the bone. Tissues can also form in a rather exaggerated manner, as in cases of macrognathia. The latter corresponds to the simple type dysplasia, as it affects one tissue only. Dysplasia might be grouped as simple, as well as hamartomas and heteroplasia. 

At the final stage of the fetal period, when everything is nearly completely formed but not yet definite, anatomical parts can be put under pressure, tension, constriction and lack of movement inside the uterus. This might lead to deformities of specific parts, particularly concerning their final shape. Limbs might become rather crooked, feet improperly positioned, and the skull might become flattened. The aforementioned developmental disorders are known as deformities. Both dysplasia and deformities are disorders of the fetal period, since histogenesis has been jeopardized. 

## HOW LONG DOES EACH DISORDER LAST?

Developmental disorders and the time for intrauterine growth can be determined and predictable (Fig 1). However, after birth, many tissues remain at embryonic and fetal stages; in other words, under organogenesis and histogenesis, respectively. 

A good example is one’s teeth, many of which have not had tooth buds formed; that is, they remain as embryos and fetuses. Likewise, some parts of the jaws are under development towards formation of adult craniomandibular tissues.

Nevertheless, a few cases have external agents affecting formation, for instance:

1) When thumb-sucking affects mandibular growth, the result is deformity. 2) When there is overgrowth of the mandible relative to the maxilla, macrognathia accounts for simple dysplasia. 3) In many cases, disproportionate relationship between the jaws due to incorrect position, rather than inconsistent size, accounts for deformity. 4) Cleft lip is caused between the fourth and eighth week of intrauterine life, being considered an anomaly or malformation.

A few more examples: 1) Partial anodontia or supernumerary teeth are anomalies or malformations of the tooth. 2) Root dilaceration is a deformity. 3) Fluorosis and enamel white spots are not carious lesions, but rather hypoplastic anomalies. 4) Invaginated teeth or dens in dente is a dysplasia of dental tissues. 

In short: some tissues and organs have their embryonic and fetal periods started and/or extended beyond classic periods, even after birth. A human body is considered completely formed after the age of 22 to 24 years.

## CAN MAXILLA BE HYPOPLASTIC OR WOULD IT BE ATROPHIC? OR WHAT IS THE DIFFERENCE BETWEEN DEVELOPMENTAL DISORDERS AND CELL GROWTH DISORDERS?

Once development has ceased, with completely mature tissues, there might still occur alterations in the features of tissues and organs due to cell proliferation that has been pathologically modified. However, those disorders become known as cell growth disorders.

Cell growth disorders affect adult and mature tissues which, thus, increase in size and number; in addition to changing the morphology of adult cells. This is because they affect the cell cycle of renewal and adaptation of tissues to functional demands.

Cell growth disorders include hyperplasia, hypertrophy, metaplasia, and neoplasms. Hyperplasia, hypertrophy and metaplasia arise with a view to adapting adult tissues to new functional demands. On the other hand, neoplasms result from loss of mechanisms that control cell proliferation. Whether benign or malign, they are undesirable.

Atrophy is not a cell growth disorder, much less a developmental disorder. Atrophy is a reduction in volume of tissues and organs due to a decrease in volume and number of cells. This is caused by autophagy, a process that allows degradation of organelles and other structures, a real mechanism of involution and tissue degradation with functional as well as structural consequences previously referred to as degenerative.

Upon the assertion that one’s maxilla has become atrophic, the following must be taken into consideration: Has it ever been normal in size? Has it ever been normal in volume and shape? If the maxilla has never been normal in size, it has never become atrophic; it is rather a hypoplastic maxilla. Atrophy is not a developmental disorder, but a process of regression or involution of tissues or organs with decreased function. Importantly, it is induced by numerous causes.

Both maxilla and mandible might be atrophic in the event of tooth loss. At those sites, or at the entire dental arch, there will be partial or total loss of maxillary and/or mandibular alveolar processes, in addition to reduction in volume and size of the jaws. The bone extension also known as alveolar process exists to provide teeth support - without them, it disappears. Atrophy of the jaws will have consequences, such as loss of masticatory function, exposure of neural structures, and pneumatization via maxillary sinus.

**Figure 2:**
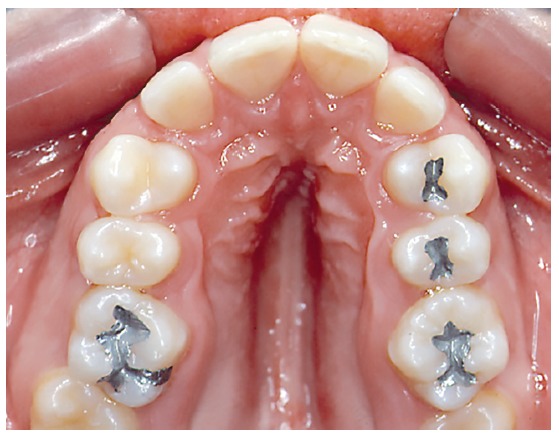
Small and high palate with narrow deformed dental arch and alveolar process in hypoplastic maxilla.

Hypoplasia, in turn, is a developmental disorder, comprising the group of anomalies or malformations, characterized by incomplete development of a tissue or organ at the organogenesis stage (Fig 2). Small/underdeveloped maxilla or mandible might be referred to as hypoplastic. However, in most cases, the term maxillary or mandibular hypoplasia is exchanged by micrognathia due to being more specific and equally appropriate from a conceptual point of view. Micrognathia can be defined as small maxilla or mandible due to not only hypoplasia, which means incomplete or smaller size, but also disorder or insufficient maxillary growth. 

The assertion that a maxilla is small sized induces us to think it has ever been normal, which is inaccurate. Therefore, size has never been “reduced” or decreased. Should there be tooth loss, atrophy of the jaws will partially or completely occur.

## CAN MAXILLA BE ATRESIC OR WOULD IT BE HYPOPLASTIC ? THE CONCEPT OF ATRESIA!

Comprising the groups of anomalies or malformations caused by incomplete development of an organ and/or tissue, it is the term atresia. The latter stands for insufficient or lack of development of lumina in hollow organs, such as the digestive tube and excretory ducts, of which embryonic outlines had been previously solid and then became hollow. Absence of such lumen or gap occludes and blocks passage through organs.

Despite largely used, the term “atresic maxilla” refers to small maxillary size or volume. This means the most appropriate is “hypoplastic maxilla” (Fig 2). 

Maxilla is not a hollow organ; it has no lumen to be blocked, which would characterize atresia. Stenosis is another term used to represent local narrowing of lumina that, at a specific site, are incomplete in dimension.

“Hypoplastic maxilla” is more appropriate. Let us recall the concept of hypoplasia: deficient development of an organ or tissue; however, without completely damaging function. They are organs that are deficient in size, but allow individuals to live a quite normal life. The condition can be corrected by surgical, orthopedic and/or orthodontic treatment. 

## FINAL CONSIDERATIONS

“Atresic” and “atrophic” seem to be improper terms when referring to maxilla and mandible with deficient growth compared to development that is satisfactory enough to reach normal size. Although some might see it as vice of language, the use of “atresic” is hard to understand. This is because, from a conceptual point of view, it goes against the nomenclature used to study and report human developmental disorders.

To identify smaller maxilla and mandible, the most proper and accurate term is hypoplastic maxilla or mandible (Fig 2). This is because “atresia” stands for an anomaly related to lumen blockage in hollow organs, which is not the case for neither maxilla nor mandible. Hypoplastic maxilla or mandible can be properly and specifically referred to as micrognathia.
